# 3,5-Dichloro­phenyl 4-methyl­benzoate

**DOI:** 10.1107/S1600536808022277

**Published:** 2008-07-19

**Authors:** B. Thimme Gowda, Sabine Foro, K. S. Babitha, Hartmut Fuess

**Affiliations:** a Department of Chemistry, Mangalore University, Mangalagangotri 574 199, Mangalore, India; b Institute of Materials Science, Darmstadt University of Technology, Petersenstrasse 23, D-64287 Darmstadt, Germany

## Abstract

The structure of the title compound, C_14_H_10_Cl_2_O_2_, resembles those of 3-chloro­phenyl 4-methyl­benzoate, 2,6-dichloro­phenyl 4-methyl­benzoate and 2,4-dichloro­phenyl 4-methyl­benzoate, with similar bond parameters. The dihedral angle between the benzene and benzoyl rings is 48.81 (6)°.

## Related literature

For related literature, see: Gowda *et al.* (2007 ▶, 2008*a* ▶,*b* ▶); Nayak & Gowda (2008 ▶).
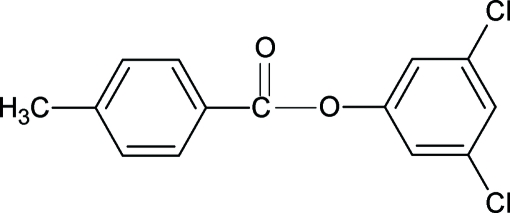


## Experimental

### 

#### Crystal data

C_14_H_10_Cl_2_O_2_
*M* *_r_* = 281.12
Monoclinic, 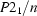

*a* = 3.9273 (6) Å
*b* = 28.412 (4) Å
*c* = 11.705 (1) Å
β = 94.06 (1)°
*V* = 1302.8 (3) Å^3^
*Z* = 4
Mo *K*α radiation
μ = 0.49 mm^−1^
*T* = 299 (2) K
0.48 × 0.40 × 0.08 mm

#### Data collection

Oxford Diffraction Xcalibur diffractometer with a Sapphire CCD detector
Absorption correction: multi-scan (*CrysAlis RED*; Oxford Diffraction, 2007 ▶) *T*_min_ = 0.800, *T*_max_ = 0.962
7613 measured reflections
2621 independent reflections
1755 reflections with *I* > 2σ(*I*)
*R*_int_ = 0.025

#### Refinement

*R*[*F* ^2^ > 2σ(*F* ^2^)] = 0.040
*wR*(*F* ^2^) = 0.139
*S* = 1.11
2621 reflections
184 parameters
H atoms treated by a mixture of independent and constrained refinement
Δρ_max_ = 0.32 e Å^−3^
Δρ_min_ = −0.24 e Å^−3^

### 

Data collection: *CrysAlis CCD* (Oxford Diffraction, 2004 ▶); cell refinement: *CrysAlis RED* (Oxford Diffraction, 2007 ▶); data reduction: *CrysAlis RED*; program(s) used to solve structure: *SHELXS97* (Sheldrick, 2008 ▶); program(s) used to refine structure: *SHELXL97* (Sheldrick, 2008 ▶); molecular graphics: *PLATON* (Spek, 2003 ▶); software used to prepare material for publication: *SHELXL97*.

## Supplementary Material

Crystal structure: contains datablocks I, global. DOI: 10.1107/S1600536808022277/bx2159sup1.cif

Structure factors: contains datablocks I. DOI: 10.1107/S1600536808022277/bx2159Isup2.hkl

Additional supplementary materials:  crystallographic information; 3D view; checkCIF report

## Figures and Tables

**Fig. 1.**
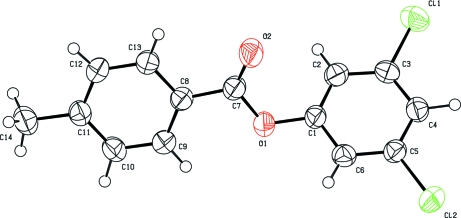
Molecular structure of the title compound, showing the atom labelling scheme. The Displacement ellipsoids are drawn at the 50% probability level. The H atoms are represented as small spheres of arbitrary radii.

**Fig. 2.**
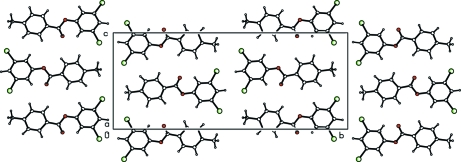
Molecular packing of the title compound.

## supplementary crystallographic information

### Comment

In the present work, as part of a study of the substituent effects on the structures of chemically and industrially significant compounds (Gowda *et al.*, 2007, 2008*a, b*), the structure of 3,5-dichlorophenyl 4-methylbenzoate (35DCP4MeBA) has been determined. The structure of 35DCP4MeBA (Fig. 1) resembles those of 3-chlorophenyl 4-methylbenzoate (3CP4MeBA)(Gowda *et al.*, 2008*b*), 2,6-dichlorophenyl 4-methylbenzoate (26DCP4MeBA)(Gowda *et al.*, 2008*a*), 2,4-dichlorophenyl 4-methyl benzoate (24DCP4MeBA) and other aryl benzoates (Gowda *et al.*, 2007). The bond parameters in 35DCP4MeBA are similar to those in 3CP4MeBA, 26DCP4MeBA, 24DCP4MeBA and other benzoates. The dihedral angle between the benzene and benzoyl rings in 35DCP4MeBA is 48.81 (6)°, compared to the values of 71.75 (7)° in 3CP4MeBA (Gowda *et al.*, 2008*b*), 77.97 (9)° in 26DCP4MeBA (Gowda *et al.*, 2008*a*) and 48.13 (5)° in 24DCP4MeBA) (Gowda *et al.*, 2007). The molecular packing in the crystal structure of 35DCP4MeBA is shown in Fig. 2.

### Experimental

The title compound was prepared according to a literature method of Nayak & Gowda (2008). The purity of the compound was checked by determining its melting point. It was characterized by recording its infrared and NMR spectra (Nayak & Gowda, 2008). Single crystals of the title compound used for X-ray diffraction studies were obtained by a slow evaporation of an ethanolic solution at room temperature.

### Refinement

The H atoms of the methyl group were positioned with idealized geometry using a riding model with C—H = 0.96 Å. The other H atoms were located in difference map, and its positional parameters were refined freely [C—H = 0.89 (3)–1.02 (3) Å. All H atoms were refined with isotropic displacement parameters (set to 1.2 times of the *U*_eq_ of the parent atom).

### Crystal data

**Table:**

C_14_H_10_Cl_2_O_2_	*F*_000_ = 576
*M**_r_* = 281.12	*D*_x_ = 1.433 Mg m^−^^3^
Monoclinic, *P*2_1_/*n*	Mo *K*α radiation λ = 0.71073 Å
Hall symbol: -P 2yn	Cell parameters from 3406 reflections
*a* = 3.9273 (6) Å	θ = 2.3–27.9º
*b* = 28.412 (4) Å	µ = 0.49 mm^−^^1^
*c* = 11.705 (1) Å	*T* = 299 (2) K
β = 94.06 (1)º	Plate, colourless
*V* = 1302.8 (3) Å^3^	0.48 × 0.40 × 0.08 mm
*Z* = 4	

### Data collection

**Table:**

Oxford Diffraction Xcalibur diffractometer with a Sapphire CCD detector	2621 independent reflections
Radiation source: fine-focus sealed tube	1755 reflections with *I* > 2σ(*I*)
Monochromator: graphite	*R*_int_ = 0.025
*T* = 299(2) K	θ_max_ = 26.4º
Rotation method data acquisition using ω and φ scans	θ_min_ = 2.3º
Absorption correction: multi-scan(CrysAlis RED; Oxford Diffraction, 2007)	*h* = −4→4
*T*_min_ = 0.800, *T*_max_ = 0.962	*k* = −35→34
7613 measured reflections	*l* = −14→14

### Refinement

**Table:**

Refinement on *F*^2^	Secondary atom site location: difference Fourier map
Least-squares matrix: full	Hydrogen site location: inferred from neighbouring sites
*R*[*F*^2^ > 2σ(*F*^2^)] = 0.040	H atoms treated by a mixture of independent and constrained refinement
*wR*(*F*^2^) = 0.139	*w* = 1/[σ^2^(*F*_o_^2^) + (0.0783*P*)^2^] where *P* = (*F*_o_^2^ + 2*F*_c_^2^)/3
*S* = 1.11	(Δ/σ)_max_ = 0.001
2621 reflections	Δρ_max_ = 0.32 e Å^−^^3^
184 parameters	Δρ_min_ = −0.24 e Å^−^^3^
Primary atom site location: structure-invariant direct methods	Extinction correction: none

### Special details

**Table:**

Experimental. CrysAlis RED, Oxford Diffraction Ltd., 2007 Empirical absorption correction using spherical harmonics, implemented in SCALE3 ABSPACK scaling algorithm.
Geometry. All e.s.d.'s (except the e.s.d. in the dihedral angle between two l.s. planes) are estimated using the full covariance matrix. The cell e.s.d.'s are taken into account individually in the estimation of e.s.d.'s in distances, angles and torsion angles; correlations between e.s.d.'s in cell parameters are only used when they are defined by crystal symmetry. An approximate (isotropic) treatment of cell e.s.d.'s is used for estimating e.s.d.'s involving l.s. planes.
Refinement. Refinement of *F*^2^ against ALL reflections. The weighted *R*-factor *wR* and goodness of fit *S* are based on *F*^2^, conventional *R*-factors *R* are based on *F*, with *F* set to zero for negative *F*^2^. The threshold expression of *F*^2^ > σ(*F*^2^) is used only for calculating *R*-factors(gt) *etc*. and is not relevant to the choice of reflections for refinement. *R*-factors based on *F*^2^ are statistically about twice as large as those based on *F*, and *R*- factors based on ALL data will be even larger.

### Fractional atomic coordinates and isotropic or equivalent isotropic displacement parameters (Å^2^)

**Table:**

	*x*	*y*	*z*	*U*_iso_*/*U*_eq_	
C1	0.4042 (6)	0.15541 (8)	−0.1194 (2)	0.0418 (6)	
C2	0.2829 (6)	0.13459 (9)	−0.0235 (2)	0.0444 (6)	
H2	0.200 (6)	0.1519 (9)	0.035 (2)	0.053*	
C3	0.2772 (6)	0.08640 (9)	−0.01881 (19)	0.0419 (6)	
C4	0.3809 (6)	0.05839 (9)	−0.1061 (2)	0.0442 (6)	
H4	0.373 (5)	0.0228 (10)	−0.0960 (19)	0.053*	
C5	0.4990 (6)	0.08058 (9)	−0.20047 (19)	0.0413 (6)	
C6	0.5140 (6)	0.12902 (9)	−0.2088 (2)	0.0432 (6)	
H6	0.583 (6)	0.1444 (9)	−0.270 (2)	0.052*	
C7	0.5453 (6)	0.23217 (9)	−0.0486 (2)	0.0450 (6)	
C8	0.4837 (6)	0.28221 (8)	−0.0721 (2)	0.0405 (6)	
C9	0.3278 (7)	0.29931 (10)	−0.1734 (2)	0.0492 (7)	
H9	0.260 (7)	0.2789 (10)	−0.231 (2)	0.059*	
C10	0.2734 (7)	0.34658 (10)	−0.1884 (2)	0.0506 (7)	
H10	0.164 (6)	0.3558 (9)	−0.263 (2)	0.061*	
C11	0.3722 (6)	0.37871 (9)	−0.1031 (2)	0.0437 (6)	
C12	0.5333 (7)	0.36165 (10)	−0.0028 (2)	0.0477 (7)	
H12	0.607 (6)	0.3843 (9)	0.049 (2)	0.057*	
C13	0.5880 (7)	0.31429 (9)	0.0140 (2)	0.0464 (7)	
H13	0.689 (6)	0.3038 (9)	0.079 (2)	0.056*	
C14	0.3049 (8)	0.43029 (10)	−0.1188 (3)	0.0598 (8)	
H14A	0.5179	0.4468	−0.1197	0.072*	
H14B	0.1808	0.4417	−0.0567	0.072*	
H14C	0.1729	0.4354	−0.1899	0.072*	
O1	0.3978 (5)	0.20416 (6)	−0.13445 (15)	0.0544 (5)	
O2	0.7075 (5)	0.21609 (6)	0.03319 (17)	0.0650 (6)	
Cl1	0.13414 (18)	0.05912 (3)	0.10169 (6)	0.0602 (3)	
Cl2	0.6296 (2)	0.04648 (2)	−0.31227 (6)	0.0605 (3)	

### Atomic displacement parameters (Å^2^)

**Table:**

	*U*^11^	*U*^22^	*U*^33^	*U*^12^	*U*^13^	*U*^23^
C1	0.0491 (15)	0.0345 (15)	0.0402 (13)	0.0037 (11)	−0.0078 (11)	−0.0019 (10)
C2	0.0466 (15)	0.0452 (16)	0.0408 (14)	0.0089 (12)	−0.0020 (11)	−0.0037 (11)
C3	0.0391 (13)	0.0472 (16)	0.0389 (13)	0.0018 (11)	0.0000 (10)	0.0042 (11)
C4	0.0464 (15)	0.0382 (15)	0.0473 (15)	0.0007 (11)	−0.0014 (11)	0.0021 (11)
C5	0.0436 (14)	0.0397 (14)	0.0402 (13)	0.0001 (11)	−0.0003 (10)	−0.0035 (11)
C6	0.0460 (14)	0.0445 (16)	0.0384 (13)	−0.0056 (12)	−0.0014 (11)	0.0015 (11)
C7	0.0496 (15)	0.0404 (15)	0.0443 (14)	0.0022 (12)	−0.0024 (12)	−0.0045 (11)
C8	0.0396 (13)	0.0399 (15)	0.0414 (13)	0.0021 (11)	0.0000 (10)	−0.0020 (11)
C9	0.0616 (17)	0.0426 (16)	0.0425 (15)	−0.0007 (13)	−0.0017 (12)	−0.0020 (12)
C10	0.0597 (17)	0.0440 (17)	0.0468 (15)	0.0019 (12)	−0.0043 (13)	0.0081 (12)
C11	0.0448 (14)	0.0384 (15)	0.0493 (15)	0.0026 (11)	0.0122 (11)	0.0029 (11)
C12	0.0562 (16)	0.0397 (16)	0.0470 (15)	−0.0010 (12)	0.0016 (12)	−0.0072 (12)
C13	0.0540 (16)	0.0407 (16)	0.0433 (15)	0.0034 (12)	−0.0059 (12)	−0.0013 (12)
C14	0.0715 (19)	0.0452 (18)	0.0641 (19)	0.0082 (14)	0.0148 (14)	0.0106 (13)
O1	0.0802 (13)	0.0341 (11)	0.0465 (10)	0.0014 (9)	−0.0120 (9)	−0.0001 (8)
O2	0.0828 (14)	0.0442 (11)	0.0635 (12)	0.0109 (10)	−0.0274 (11)	−0.0019 (9)
Cl1	0.0681 (5)	0.0635 (5)	0.0504 (4)	−0.0026 (3)	0.0136 (3)	0.0100 (3)
Cl2	0.0806 (5)	0.0503 (5)	0.0521 (4)	0.0015 (4)	0.0149 (3)	−0.0108 (3)

### Geometric parameters (Å, °)

**Table:**

C1—C6	1.382 (3)	C8—C9	1.383 (4)
C1—C2	1.382 (3)	C8—C13	1.398 (3)
C1—O1	1.396 (3)	C9—C10	1.369 (4)
C2—C3	1.371 (3)	C9—H9	0.91 (3)
C2—H2	0.92 (3)	C10—C11	1.387 (4)
C3—C4	1.379 (3)	C10—H10	0.98 (3)
C3—Cl1	1.737 (2)	C11—C12	1.382 (3)
C4—C5	1.381 (3)	C11—C14	1.498 (3)
C4—H4	1.02 (3)	C12—C13	1.375 (4)
C5—C6	1.382 (3)	C12—H12	0.91 (3)
C5—Cl2	1.735 (2)	C13—H13	0.89 (3)
C6—H6	0.89 (3)	C14—H14A	0.9600
C7—O2	1.202 (3)	C14—H14B	0.9600
C7—O1	1.377 (3)	C14—H14C	0.9600
C7—C8	1.465 (3)		
			
C6—C1—C2	121.8 (2)	C13—C8—C7	117.5 (2)
C6—C1—O1	116.5 (2)	C10—C9—C8	120.7 (2)
C2—C1—O1	121.5 (2)	C10—C9—H9	119.5 (18)
C3—C2—C1	117.9 (2)	C8—C9—H9	119.9 (18)
C3—C2—H2	119.8 (16)	C9—C10—C11	121.3 (3)
C1—C2—H2	122.3 (16)	C9—C10—H10	115.5 (16)
C2—C3—C4	122.7 (2)	C11—C10—H10	123.2 (16)
C2—C3—Cl1	119.06 (19)	C12—C11—C10	117.9 (2)
C4—C3—Cl1	118.25 (19)	C12—C11—C14	120.9 (2)
C3—C4—C5	117.6 (2)	C10—C11—C14	121.2 (2)
C3—C4—H4	118.3 (13)	C13—C12—C11	121.5 (3)
C5—C4—H4	124.1 (13)	C13—C12—H12	123.8 (17)
C4—C5—C6	122.0 (2)	C11—C12—H12	114.7 (17)
C4—C5—Cl2	118.88 (19)	C12—C13—C8	120.0 (2)
C6—C5—Cl2	119.10 (18)	C12—C13—H13	120.5 (17)
C5—C6—C1	118.0 (2)	C8—C13—H13	119.5 (17)
C5—C6—H6	124.0 (17)	C11—C14—H14A	109.5
C1—C6—H6	117.9 (17)	C11—C14—H14B	109.5
O2—C7—O1	122.2 (2)	H14A—C14—H14B	109.5
O2—C7—C8	126.1 (2)	C11—C14—H14C	109.5
O1—C7—C8	111.6 (2)	H14A—C14—H14C	109.5
C9—C8—C13	118.6 (2)	H14B—C14—H14C	109.5
C9—C8—C7	124.0 (2)	C7—O1—C1	118.56 (18)
			
C6—C1—C2—C3	0.6 (4)	O1—C7—C8—C13	173.3 (2)
O1—C1—C2—C3	175.7 (2)	C13—C8—C9—C10	−0.8 (4)
C1—C2—C3—C4	−1.2 (4)	C7—C8—C9—C10	178.9 (2)
C1—C2—C3—Cl1	178.75 (18)	C8—C9—C10—C11	0.0 (4)
C2—C3—C4—C5	0.9 (4)	C9—C10—C11—C12	1.1 (4)
Cl1—C3—C4—C5	−179.01 (18)	C9—C10—C11—C14	−178.5 (2)
C3—C4—C5—C6	−0.1 (4)	C10—C11—C12—C13	−1.5 (4)
C3—C4—C5—Cl2	−179.67 (18)	C14—C11—C12—C13	178.1 (2)
C4—C5—C6—C1	−0.4 (4)	C11—C12—C13—C8	0.8 (4)
Cl2—C5—C6—C1	179.14 (18)	C9—C8—C13—C12	0.3 (4)
C2—C1—C6—C5	0.2 (4)	C7—C8—C13—C12	−179.3 (2)
O1—C1—C6—C5	−175.2 (2)	O2—C7—O1—C1	7.5 (4)
O2—C7—C8—C9	172.5 (3)	C8—C7—O1—C1	−173.6 (2)
O1—C7—C8—C9	−6.3 (4)	C6—C1—O1—C7	−131.0 (2)
O2—C7—C8—C13	−7.9 (4)	C2—C1—O1—C7	53.6 (3)

## References

[bb1] Gowda, B. T., Foro, S., Babitha, K. S. & Fuess, H. (2007). *Acta Cryst.* E**63**, o3877.

[bb2] Gowda, B. T., Foro, S., Babitha, K. S. & Fuess, H. (2008*a*). *Acta Cryst.* E**64**, o843. 10.1107/S1600536808009616 PMC2961242 21202331

[bb3] Gowda, B. T., Foro, S., Babitha, K. S. & Fuess, H. (2008*b*). *Acta Cryst.* E**64**, o1390. 10.1107/S1600536808019351 PMC2962023 21203110

[bb4] Nayak, R. & Gowda, B. T. (2008). *Z. Naturforsch. Teil A*, **63** In the press.

[bb5] Oxford Diffraction (2004). *CrysAlis CCD* Oxford Diffraction Ltd, Köln, Germany.

[bb6] Oxford Diffraction (2007). *CrysAlis RED* Oxford Diffraction Ltd, Köln, Germany.

[bb7] Sheldrick, G. M. (2008). *Acta Cryst.* A**64**, 112–122. 10.1107/S0108767307043930 18156677

[bb8] Spek, A. L. (2003). *J. Appl. Cryst.* **36**, 7–13.

